# Activation of Adenosine 2A receptor inhibits neutrophil apoptosis in an autophagy-dependent manner in mice with systemic inflammatory response syndrome

**DOI:** 10.1038/srep33614

**Published:** 2016-09-20

**Authors:** Yang-Wuyue Liu, Ting Yang, Li Zhao, Zhenhong Ni, Nan Yang, Fengtian He, Shuang-Shuang Dai

**Affiliations:** 1 Department of Biochemistry and Molecular Biology, Third Military Medical University, Chongqing 400038, PRC; 2 Molecular Biology Center, State Key Laboratory of Trauma, Burn, and Combined Injury, Daping Hospital, Third Military Medical University, Chongqing 400042, PRC

## Abstract

Systemic inflammatory response syndrome (SIRS) is an overwhelming whole body inflammation caused by infectious diseases or sterile insults. Neutrophils are the dominant participants during inflammation, and their survival and death determine the initiation as well as resolution of SIRS. Apoptosis and autophagy are two fundamental cellular processes that modulating cell fate, but their correlation and regulators in neutrophils under SIRS condition have not been elucidated. In this study, we demonstrated that high dose of LPS induced both apoptosis and autophagy of neutrophils in a mouse SIRS model and LPS-stimulated neutrophils *in vitro*. Moreover, we found that the adenosine 2A receptor (A2AR), a known anti-inflammatory G protein-coupled receptor (GPCR), could inhibit LPS-induced neutrophil apoptosis by suppressing the LPS-induced autophagy. Activation of A2AR suppressed LPS-induced autophagy by inhibiting the ROS-JNK pathway as well as promoting GPCR βϒ subunit–AKT signaling. The A2AR-inhibited autophagy suppressed apoptosis of neutrophils by blocking caspase8, caspase3 and PARP signaling. These findings not only increase our understandings of neutrophils’ fate and function in response to systemic inflammation, but also identify a novel anti-inflammatory role of A2AR in modulating neutrophils’ survival during inflammation.

Systemic inflammatory response syndrome (SIRS) is defined as a massive inflammatory reaction caused by infection or sterile complications, such as severe trauma, extensive burns, and shock^1^. Roger C Bone divided the SIRS into 5 phases: local response, initial systemic response, massive systemic inflammatory response, compensated anti-inflammatory response and immunologic dissonance^2^. During these phases, the balance of inflammatory mediators and the survival or death of immune cells drive SIRS development. Neutrophils, as the most abundant peripheral circulating immune cells, constitute the first-line defensor against invading pathogens and play indispensable roles in the early stages of inflammation and immune responses. Besides the neutrophil functions including phagocytosis, degranulation, cytokine release and neutrophil extracellular traps (NETs) formation, the lifespan of neutrophils is also important in SIRS because it is strongly associated with the initiation as well as the resolution of inflammatory responses^3^,^4^,^5^. Apoptosis is a critical process to regulate neutrophil lifespan and has been shown to be related to the severity of SIRS in the clinic^6^. However, the significance of neutrophil apoptosis may have different meanings depending on the situations. If the local infection is present, neutrophil migration to the bacterial invasion site starts immediately and phagocytic process should proceed. It is well known that prolongation of life span of neutrophils migrating from blood stream to inflammation sites occurs. Under this condition, inhibition of physiological apoptotic process may be present in specific population of migrating neutrophils with several days life span and this inhibition appears to be beneficial. Thus, identifying some inhibitors of neutrophil apoptosis in the initial phase of SIRS may provide novel targets for suppressing SIRS development.

In recent researches, apoptosis is reported to have some closely relationship with autophagy, a well-conserved, essential, intracellular degradation process known to modulate protein and organelle turnover^7^. Autophagy is generally believed to be a “double-edged sword” that controls the fate of cells because it not only helps cell survive during nutrient deprivations or other stresses, but also jeopardizes cells and induces cell death. In some cells, such as breast cancer cells^8^, leukemic cells^9^ and neurons^10^, autophagy and apoptosis cooperate to regulate cell survival and death. However, whether apoptosis and autophagy of neutrophils in SIRS are positivlye or negatively correlated, and whether some molecules can regulate the two essential processes synergistically are not clear.

A2AR, an adenosine receptor, has been shown to be a promising anti-inflammatory molecule. In several animal models of inflammatory injuries including acute lung injury, ischemic kidney injury and sepsis^11^,^12^,^13^, activation of A2AR is found to inhibit the release of inflammatory mediators, phagocytosis, dagraluation and oxdative burst, which is beneficial for individual to avoid the over-damage induced by inflammation^14^. Of note, previous studies demonstrated the anti-inflammation effect of A2AR on the regulation of neutrophils’ functions, including decrease the expression and stickiness of adhesion molecules alpha 4/beta 1 integrin^15^; prevent the expression and release of TNF-alpha, MIP-1alpha/CCL3, MIP-1beta/CCL4, MIP-2alpha/CXCL2, and MIP-3alpha/CCL20^16^. Recently, some researchers found that A2AR could delay human neutrophil’s apoptosis significantly under normal condition^17^ and provide cardioprotection by downreguting autophagy^18^. However, it is not clear whether A2AR affect neutrophil autophagy and apoptosis under inflammatory condition.

Thus, in the present study, we investigated the effect of A2AR on neutrophil apoptosis and autophagy, and their relationship in an LPS-induced SIRS model, and deciphered the detailed molecular mechanisms in isolated murine neutrophils.We found that A2AR inhibited apoptosis of neutrophils in an autophagy-dependent manner both *in vivo* and *in vitro*. Activation of A2AR suppressed LPS-induced autophagy by inhibiting the ROS-JNK pathway as well as promoting GPCR βϒ subunit–AKT signaling. The A2AR-inhibited autophagy suppressed apoptosis of neutrophils by blocking caspase8, caspase3 and PARP signaling. These findings not only increase our understandings of neutrophils’ fate and function in response to systemic inflammation, but also identify a novel role of A2AR in modulating neutrophils’ survival during inflammation.

## Results

### Knockout of A2AR exacerbated SIRS severity, deteriorated apoptosis and autophagy of bone marrow cells and neutrophils in LPS-induced SIRS models

**Figure 1:**
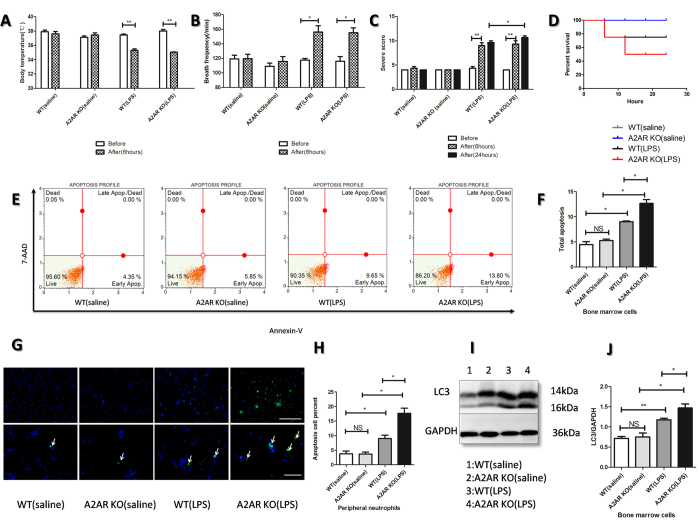
Detections of A2AR effect on apoptosis and autophagy of bone marrow cells and peripheral neutrophils in LPS-induced SIRS model. (**A–C**) Evaluations of SIRS related indexes including rectal temperature (**A**), breath frequency (**B**) after LPS intraperitoneal injection (11.25 million EU/kg, 10 mg/Kg) for 6 hours. The SIRS severity scores (**C**) were counted after LPS injection for 6 hours and 24 hours. Eaqual saline was injected into WT/A2AR KO mice as control. White bars represent before injection and gilled bars represent after injection for 6 hours, black bars represent severtiy score after 24 hours development. (**D**) Survival rates of each group. Each group contained 5 mice treated with intended conditions. The mortality of each group was counted until 24 hours’ development. (**E**) Flow cytometry analyse of apoptosis cells on bone marrow cells which were harvested after LPS injection for 24 hours. The harvested bone marrow cells were stained with AnnexinV/7-AAD and analyzed using MUSE cell analyzer. (**F)** The quantification of total apoptotic bone marrow cells in (**E**). (**G**) Detection of apoptosis neutrophils isolated from mice peripheral blood. The sample slides were stained by Apoptag flurorescein to detect the apoptosis cells, as the white arrows show. The nucleus were stained by DAPI. The upper white line represents 100 μm and the lower white line represents 50 μm. (**H**) The counted proportions of total apoptotic cells in mice peripheral neutrophils in (**G**). (**I**) Assays of autophagy related molecule LC3 by WB. The protein lysis were achieved from isolated bone marrow cells of above four groups. The ratio of LC3/GAPDH was calculated to reflect the level of autophagy. (**J**) The arbitrary units were shown as the densitometry ratio of LC3 compared with GAPDH. Each mice group (except the survival experiment in **D** contained 5 animals) contained 3 animals which were with similar weight and age. Data represents the mean ± SEM for three independent experiments. (*p < 0.05 between the two groups; **p < 0.01 between the two groups, NS, no significant difference between the two groups, Student’s t test).

To determine the effect of A2AR on SIRS, we utilized the LPS injection to achieve murine SIRS model as previously described^19^. The SIRS model was sucessfully achieved and evaluated by three indexes (body temperature, breath frequency. and SIRS severity score) at 6 h-post LPS injection. As shown in Fig. 1, both WT and A2AR KO mice could be successfully induced to be SIRS model with the decreased body temperature (Fig. 1A), increased breath frequency (Fig. 1B) as well as high score compared with saline group (Fig. 1C). Even though there were no significant differences of SIRS severity score between WT and A2AR KO at 6 hours,while the score in A2AR KO group was higher than WT group at 24 hours, the mortality in LPS treated A2AR KO was higher compared with LPS treated WT group (Fig. 1D), which indicates that A2AR is an important receptor involved in anti-SIRS. After 24 hours’ development, we isolated bone marrow cells (BMs) and peripheral neutrophil (PMNs) respectively to assess the apoptosis and autophagy level of bone marrow cells and peripheral neutrophils. The data showed that LPS induced BMs’ apoptosis and A2AR deficiency enhanced this apoptosis (Fig. 1E,F). The results in PMNs was consistent with BMs, which was detected by apoptag immunofluorescence (Fig. 1G,H). At the meantime, we found LPS also stimulated autophagy in BMs and abortion of A2AR could enhance LPS-induced autophagy significantly (Fig. 1I,J). This consistency of A2AR effects on altered autophagy and apoptosis levels indicated that apoptosis and autophagy of neutrophil in SIRS may have a synergistic or positive correlation, and A2AR is a potential modulator of neutrophil fate by affecting apoptosis and autophagy.

### LPS induced apoptosis and autophagy of neutrophils *in vitro*

**Figure 2:**
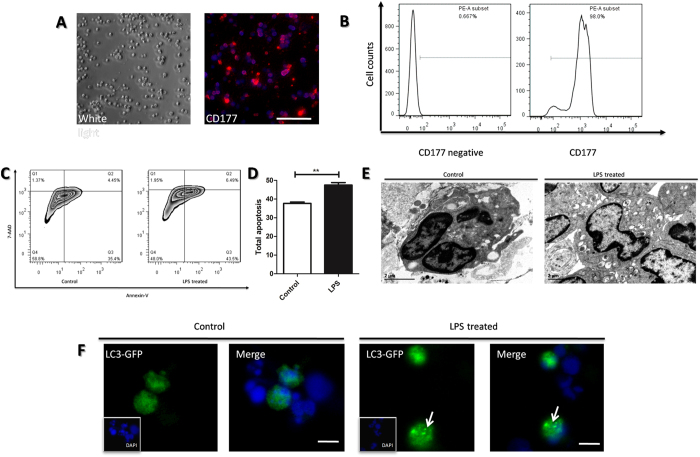
Assays of apoptosis and autophagy in primary-isolated neutrophils induced by LPS. (**A**) The white light and immunofluorescence image of primary neutrophils isolated from mice bone marrow. The neutrophils were marked by CD177 and then incubated with Alexa Fluor^®^ 555. The nucleus were stained by DAPI. White bar represents 50 μm. (**B)** The qualification of CD177 positive cells in total isolated cells of (**A**) were gained through single channel flow cytometry. The CD177 negative group was incubated with PBS instead of primary antibody. (**C)** Isolated neutrophils were treated with or without LPS (1 μg/ml) and subsequently stained with AnnexinV/7-AAD and analyzed apoptosis using MUSE cell analyzer. **(D)** The qualification of apoptic cells in (**C**). **(E)** Autophagysome in neutrophils treated with or without LPS assayed by transmission electron microscope. Scale bar: 2 μm. (**F)** The fluorescence images of GFP-LC3 puncture (which occurred upon autophagy induction, as the white arrows show). The neutrophil-like cell lines, DMSO-treated HL-60 cell lines were transfected with GFP-LC3 plasmids, treated with or without LPS (1 μg/ml) stimulation for 2 hours and observed by fluorescence microscope.The nucleus was stained by DAPI. White bar represents 10 μm.

To further confirm the relationship between autophagy and apoptosis of neutrophil in SIRS, murine neutrophils were freshly isolated and stimulated with LPS, which could mimic SIRS condition *in vitro*. The purity of the isolated neutrophils was confirmed with immunofluorescence of the mature neutrophil marker CD177, and around 95% to 98% of the cells were positive (Fig. 2A,B). MUSE cell analysis demonstrated that LPS significantly induced apoptosis of neutrophils (Fig. 2C,D). Similarly, electron microscopy images showed that the LPS treatment also markedly resulted in autophagy of neutrophils, which was identified by the neutrophil vacuolation and autophagosome formation (Fig. 2E). The LPS-induced autophagy was also confirmed in neutrophil-like cells, the HL-60 cell line, which were transfetced with LC3-GFP plasmid. The characteristic punctuate pattern of accumulated LC3–GFP, which indicated autophagic induction was also significantly increased after LPS treatment (Fig. 2F). These data suggest that LPS-induced apoptosis and autophagy have a potential positive correlation, which is consistent with the observations in the murine model of SIRS described above.

### A2AR inhibited LPS-induced apoptosis in an autophagy-dependent manner

**Figure 3:**
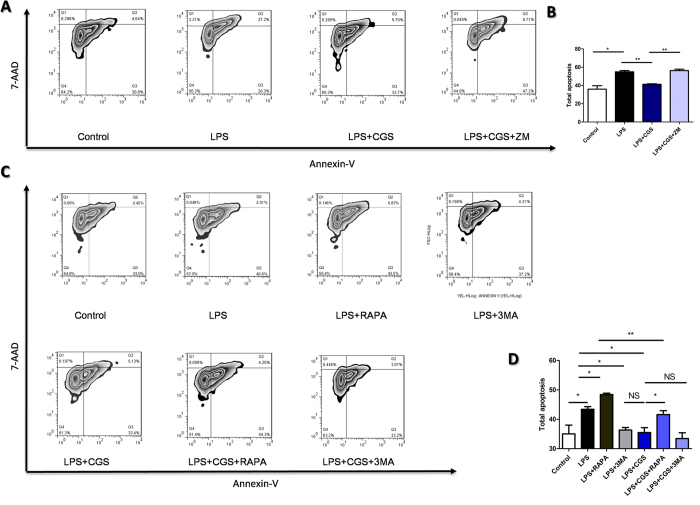
Investigation of the correlation of LPS-stimulated neutrophils’ apoptosis and autophagy in present of A2AR activation. The neutrophils’ apoptosis were modulated by A2AR in an autophagy-dependent manner. **(A)** Effect of A2AR activation on apoptosis of neutrohils treated with LPS. CGS: an A2AR selective agonist CGS21680 (0.1 μM); ZM: an A2AR selective antagonist ZM241385 (1 μM). Apoptosis was measured by MUSE cell analyzer through Annexin V-7AAD method and results were analyzed by FlowJ software. (**B)** The qualification of total apoptotic cells in (**A**) was counted. (**C)** Correlation of autophagy and the A2AR-inhibited neutrophils apoptosis induced by LPS. RAPA (50 nM) was used as autophagy inducer and 3 MA (2 mM) was used as autophagy inhibitor. Then apoptosis was measured as described above. **(D)** The qualification of total apoptotic cells in (**C**) was counted. Data represents the mean ± SEM for three independent experiments. (*p < 0.05 between the two groups; **p < 0.01 between the two groups, NS, no significant difference between the two groups, Student’s t test).

Next, we investigated the relationship between LPS-induced apoptosis and autophagy in present of A2AR activation *in vitro*. As shown in Fig. 3A,B, A2AR activation significantly suppressed apoptosis of neutrophils induced by LPS, which confirmed the results *in vivo*. Moreover, we found that the inhibitory effect of A2AR on LPS-induced neutrophil apoptosis could be mimicked by the autophagy inhibitor 3-MA and inhibited by the autophagy inducer RAPA. Combined application of 3-MA and CGS21680 did not show an increase in neutrophil apoptosis compared with that of CGS21680 treatment alone (Fig. 3C,D). These results indicate that to some extent activation of A2AR alleviating LPS-induced apoptosis may be in an autophagy-dependent manner.

### Activation of A2AR efficiently reduced LPS-induced autopahgy of neutrophils

**Figure 4:**
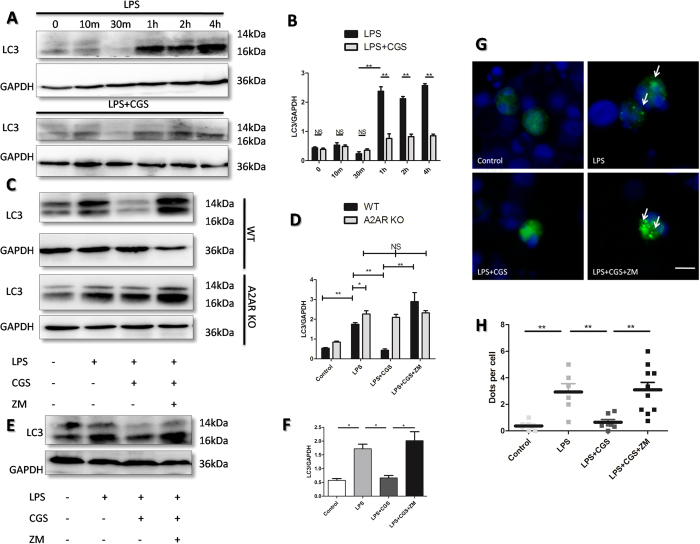
Identification of the role of A2AR on LPS-induced autopahgy in neutrophils. **(A)** Time-dependent changes of LC3 proteins in present of LPS with or without A2AR agonist CGS21680. Primary neutrophils were treated with LPS (1 μg/ml) for indicated times. Subsequently, total proteins were extracted and LC3 were detected by WB. (**B)** Quantification of LC3/GAPDH in (**A**). (**C)** Effect of A2AR on LC3expressions in LPS-induced primary neutrophils. Protein lysates of primary neutrophils isolated from WT and A2AR-KO mice were analyzed by WB. The LC3 levels were detected after indicated treatments for 2 hours, of which LPS (1 μg/ml). A2AR selective agonist CGS21680 (0.1 μM) and A2AR selective antagonist ZM241385(1 μM) were used respectively. (**D)** Quantification of LC3/GAPDH in (**C**). (**E)** WB for LC3 in neutrophil-like cell line HL-60. The HL-60 cell lines were treated with 1.25% DMSO for 4 days to acquire a neutrophil-like phenotype and the treatment was similar to that for primary neutrophils described above. (**F)** Quantification of LC3/GAPDH in (**E**). (**G)** HL-60 cells transfected with LC3-GFP plasmids were dealed with indictaed treatments as primary neutrophils. Then the cells were processed with fluorescence microscope at 40 lens. White arrows demonstrate the characteristic punctuate pattern of LC3–GFP, which occurs upon autophagic induction. White bars represents 10 μm. (**H)** The number of LC3-GFP dots per cell counted in twenty cells per condition. The nucleus was stained by DAPI. The procedures were carried out by two independent observers with high magnification fluorescence microscope. (*p < 0.05 between the two groups; **p < 0.01 between the two groups, NS, no significant difference between the two groups, Student’s t test. All the quantification of LC3B/GAPDH in WB results are represented as mean ± SEM for three independent experiments).

We further investigated the details of the A2AR-inhibited autophagy of neutrophils. We found that LPS stimulated neutrophils’ autophagy in a time-dependent manner, which could be significantly blocked by A2AR agonist CGS21680 (Fig. 4A,B). To determine whether the inhibitory effect of the A2AR agonist CGS21680 was mediated by activation of A2AR, we used the A2AR antagonist ZM241385 to confirm these results. The data showed that ZM241385 completely blocked the effect of CGS21680 on LC3 expression in LPS-treated neutrophils of WT mice while both CGS21680 and ZM241385 had no effect on LPS-treated neutrophils of A2AR KO mice (Fig. 4C,D). These results were confirmed in the neutrophil-like cell line HL-60 by assessing LC3 and LC3-GFP aggregation. (Fig. 3E–H). We demonstrated that activation of A2AR had efficient effect on LPS-induced autophagy of neutrophils.

### Inhibition of LPS-sitimulated neutrophil autophagy by A2AR was not regulated by the PKA- or PKC-dependent pathways

**Figure 5:**
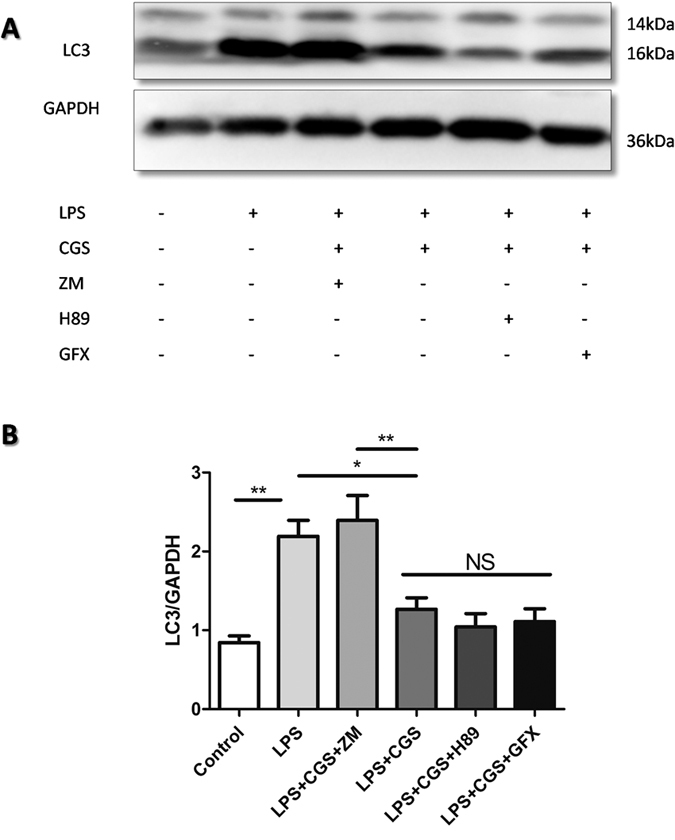
Detections of A2AR-associated autophagy pathways by PKA or PKC inhibitors. (**A)** The neutrophils were treated as figure inducated for 2 hours, then the whole proteins were extracted and LC3 were analyzed using western blot. The LPS (1 μg/ml), A2AR selective agonist CGS21680 (0.1 μM) and A2AR selective antagonist ZM241385 (1 μM) were used respectively. PKA inhibitor H89 (10 μM) and PKC inhibitor GF109203X (5 μM) were pretreated for 15 min before other drugs were added. (**B)** Quantification of LC3B/GAPDH in (**A**). The data are represented as mean ± SEM for three independent experiments. (*p < 0.05 between the two groups; **p < 0.01 between the two groups, NS, no significant difference between the two groups, Student’s t test).

Since PKA and PKC signaling are two well-known downstream of A2AR. we used the specific PKA inhibitor H89 and the PKC inhibitor GF109203X to determine whether the inhibitory effect of A2AR on LPS-induced neutrophil autophagy could be blocked. As shown in Fig. 5, the A2AR antagonist ZM241385 blocked the inhibitory effect of the A2AR agonist CGS21680 on LPS-induced neutrophil autophagy. However, H89 or GF109203X could not reverse the effect of CGS21680, indicating that A2AR regulates autophagy process in PKA- and PKC-independent pathways.

### Modulation of AKT and JNK phosphorylation was involved in the A2AR-inhibited autophagy of neutrophils treated with LPS

**Figure 6:**
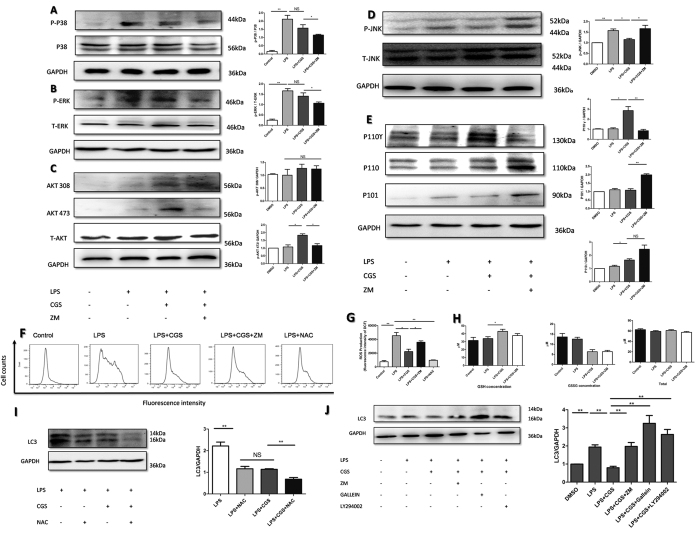
Analysis of signal pathways mediating the A2AR-inhibitory effect on LPS-induced neutrophis’ autophagy. Investigation of relative protein, ROS and GSSG during A2AR activation. (**A)** WB for p-p38 and the quantification. (**B)** WB for p-ERK and the quantification. (**C)** Western blot for p-AKT and the quantification. (**D)** WB for p-JNK and the quantification. **(E)** WB for p110γ and the quantification. (**F)** The fluorescence of DCF in neutrophils were analyzed by flow cytometry, reflecting the intracellular ROS levels. The cells were treated as indicated for 2 hours and incubated with DCFH-DA (100 μg/ml) for 30 min. The ROS inhibitor NAC (10 mM) were used as a negtive control. (**G)** Quantification of DCF fluorescence intensity in (**F**) from each group. **(H)** The antioxidents, glutathione (GSH) were detected in each neutrophils’ group. The primary neutrophils were treated as indicated for 2 hours. Then cell lysis were processed with GSH detection kit and detected through microplate spectrophotometer. Total glutathione except doubled oxidized glutathione (GSSG) were the concentrations of GSH. (**I)** Isolated neutrophil were treated as indicated for 2 hours. The ROS inhibitor NAC (10 mM) were add 15 min before other drugs were used. Then alterations in LC3 were detected by WB, in which GAPDH was used as the internal controls. (**J)** Isolated neutrophil were treated as indicated for 2 hours, during which βγ-PI3K inhibitor Gallein (10 μM) and PI3K-AKT inhibitor LY294002 (10 μM) were pretreated for 15 min. Subsequently the total proteins were extracted and probed by WB to detect the LC3 levels. The data are represented as mean ± SEM for three independent experiments. (*p < 0.05 between the two groups; **p < 0.01 between the two groups, NS, no significant difference between the two groups, Student’s t test).

As previously reported in other cells, including macrophages^20^,^21^, cardiomyocytes^22^, vascular smooth muscle cells^23^,as well as hepatocytes^24^, TLR4-p38, ROS-JNK/ERK and PI3K-AKT are three vital signaling pathways that mediate LPS-induced autophagy. This prompted us to investigate whether they are involved in the regulatory effect of A2AR on LPS-induced autophagy in neutrophils. The results showed that although LPS enhanced the expression of phosphorylated-p38 (p-p38) and phosphorylated-ERK (p-ERK) apparently, the A2AR agonist did not influence the levels of p-p38 and p-pERK significantly (Fig. 6A,B). However, activation of A2AR notably upregulated phosphorylation of AKT residue 473 and decreased phosphorylation of JNK (Fig. 6C,D). AKT phosphorylated at threonine 308 (T308) was not observed (Fig. 6C). Subsequently, to elucidate how A2AR influenced AKT and JNK activation, we detected the expressions of molecules upstream of AKT and JNK. As shown in Fig. 6C,E, in parallel with enhanced AKT phosphorylation, A2AR activation significantly increased functional P110γ. P110γ is an specific PI3K subtype which is highly expressed in neutrophil as well as the HL-60 cell line. Therefore, to validate that A2AR inhibited autophagy via the p110γ-AKT pathway, the PI3K inhibitor Gallein as well as the AKT inhibitor LY294002 were used to block inhibiton of autophagy. During this process, the A2AR antagonist ZM241385 served as a positive blocked control. The results shown that Gallein as well as LY294002 could restore the LPS- induced autophagy and even increased the levels of LC3B (Fig. 6J). This provided the hint that A2AR modulated AKT through a P110γ-associated mechanism. ROS is a well-known inducer for JNK activation. Our results demonstrated that A2AR strongly inhibited LPS-induced ROS generation, but no significant changes in GSSG and GSH were observed (Fig. 6F–H). To verify that A2AR modulates autophagy via an ROS-dependent pathway, the ROS scavenger NAC was used. We showed that NAC could inhibit LPS induced autophagy apparently as CGS did, while combination of NAC and CGS exerted stronger inhibition compared with single treatments (Fig. 6I). The data indicated that activation of A2AR could decrease ROS concentration without affecting antioxidant substances. Inhibiting the ROS level contributed to A2AR-mediated decrease in JNK phosphorylation. Taken together, these results suggest that the P110γ-AKT and ROS-JNK pathways are involved in the inhibitory effect of A2AR on LPS-induced autophagy in neutrophils.

### The A2AR-inhibited autophagy suppressed apoptosis of neutrophils by blocking caspase8, caspase3 and PARP signaling

**Figure 7:**
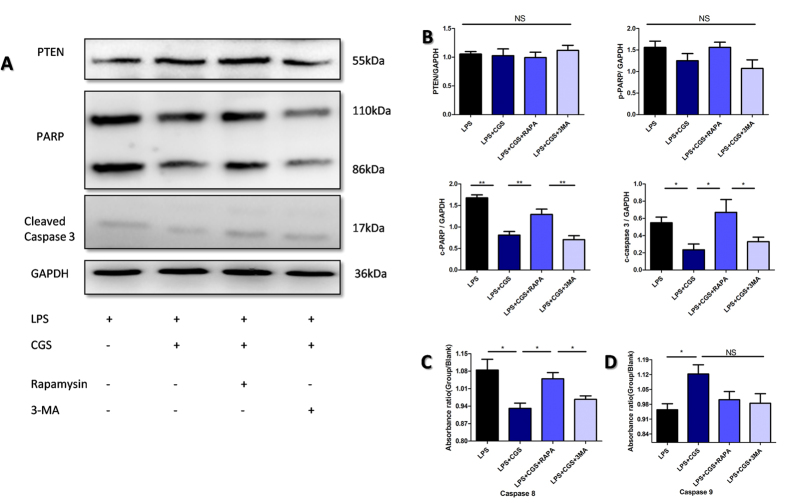
Investigation of mechanisms for A2AR inhibiting neutrophils’ apoptosis in an autophagy-dependent manner. **(A)** Isolated neutrophil were treated as indicated for 2 hours. The autophagy inducer RAPA (50 nM) and autophagy inhibitor 3 MA (2 μM) were pretreated for 15 min before LPS (1 μg/ml) and A2AR selective agonist CGS (0.1 μM) were added. Then WB were probed for freshly extracted proteins with antibodies against PTEN, PARP, cleaved caspase3 and GAPDH. **(B)** The arbitrary units for each WB results in (**A**) were shown as the densitometry ratio compared with inner control GAPDH. **(C,D)** The caspase8 and caspase9 were detected in each group of neutrophils. The primary neutrophils were treated as indicated for 2 hours. Then cell lysis were processed with caspase detection kit and analyzed using microplate spectrophotometer. Data represents the mean ± SEM for three independent experiments. (*p < 0.05 between the two groups; **p < 0.01 between the two groups, NS, no significant difference between the two groups, Student’s t test).

After elucidating how A2AR inhibited LPS-induced autophagy of neutrophils, we examined the mechanism of the A2AR-inhibited autophagy suppressed apoptosis of neutrophils. It is well known that PTEN, PARP, caspase3, 8 and 9 are major molecules involved in cell apoptosis. In the experiments, we found that although PTEN was highly expressed in isolated neutrophils, A2AR activation did not influence the PTEN expression but down-regulated cleaved PARP. cleaved-caspase 3 and caspase8 conspicuously (Fig. 7A,B). Furthermore, rapamycin, an autophagy stimulator, could restore the A2AR-inhibited cleaved PARP,cleaved caspase3 and caspase8 rather than caspase 9; while a combination of 3-MA, an autophagy inhibitor, and CGS21680 did not significantly influence these molecules compared with CGS21680 treatment alone (Fig. 7A–D). These data suggest that A2AR-inhibited autophagy suppressed apoptosis of neutrophils by blocking caspase3, caspase8 and PARP signaling.

## Discussion

Neutrophils are the major cell type involved in SIRS. Accordingly, apoptosis of neutrophils is closely associated with SIRS development. In the present study, we demonstrated that in the initial phase of SIRS, high concentration of LPS could induce the apoptosis of neutrophils while A2AR is an endogenous factor to protect neutrophils against the LPS-induced apoptosis. These results are contradictory to the previous consideration that bacterial products, like LPS, and pro-inflammatory cytokines will delay neutrophils’ apoptosis, which is an contributor to SIRS^25^,^26^. Anderson *et al*. demonstrated that neutrophils’ life span was prolonged, leading to the development of an equine SIRS model^27^; Jimenez’s observation in clinlic patients with SIRS showed that anti-apoptosis factors were present in general circulation^25^. Compared with the previous reports, our results raised two issues that need to be reconsidered in evaluating the effect of neutrophil apoptosis on SIRS: the degree of infection and the stage/time-window to detection. Since neutrophils are key innate immune cell that act as primary line of defense against invading microbial pathogens, at the initial stage, their survival and death depend on the outcome of the “battle” between the cells and pathogens. Therefore, in our study, the high concentration of LPS-induced apoptosis of peripheral neutrophils is a survival mechanism for pathogens to avoid clearence. In this view, at the acute stage, pathogens -induced neutrophil apoptosis has the potential to weaken the protective ability of the innate immune system and may contribute to the development of more severe infection, such as SIRS and sepsis. Supporting our opinion on this, Barquero-Calvo *et al*. showed that Brucella lipopolysaccharide could induce neutrophils’ death, hindering the innate function of neutrophils^28^; Liu *et al*. demonstrated that decreased survival of neutrophils leaded to higher mortality in abdominal polymicrobial sepsis^29^. Accrodingly, in our study, the effect of A2AR on delaying the LPS-induced apoptosis of neutrophils is considered to be a protective mechanism for inhibiting SIRS development. This is also consistent with the previous reports about the anti-inflammatory role of A2AR in several inflammation models^13^,^30^,^31^. However, sustained activation of neutrophils also causes an over- inflammatory response and tissue injury. In this stage, it is reasonable that delayed apoptosis of neutrophils will result in a vicious cycle leading to SIRS, which is consistent with the previous findings and opinions described above. In addition, activating A2AR does not merely mean inhibiting inflammation, since it not only inhibits adhesion and infiltration of neutrophils, suppresses inflammatory cytokine release and leukotriene synthesis^32^, but also promotes the activity of cyclooxygenase-2 and phenyl glycidyl ether 2 production^14^. Furthermore, A2AR has more potency in inhibiting neutrophil apoptosis compared with other three adenosine receptors^33^. Accordingly, the effect of high LPS on neutrophil apoptosis and the role of A2AR on the LPS-affected neutrophil apoptosis in different processes of SIRS need further investigation.

During investigating the A2AR-inhibited apoptosis of neutrophils induced by LPS, we found that the fluctuation of apoptosis was concomitant with autophagy levels. A2AR suppressed LPS-induced autophagy of neutrophils and this suppression mediated the inhibitory effect of A2AR on LPS-induced neutrophils’ apoptosis. Previously, autophagy in neutrophils was reported to be closely related to neutrophils’ immune activity, cytokine secretion, as well as extracellular trap formation in inflammatory models^34^,^35^,^36^. Here, we first demonstrated that LPS-induced autophagy of neutrophils could mediate their apoptosis. The LPS-induced autophagy has been also found in immune cells such as macrophages which were also tightly connected with their apoptosis and survival^20^,^21^. Therefore, our finding about autophagy–induced apoptosis in neutrophils provides a novel evidence and potential for autophagy in participating immune response. Furthormore, inhibition of neutrophils’ apoptosis by A2AR in an autophagy-dependent manner indicates more potential of A2AR to regulate neutrophils’ function and inflammation.

Previous investigations presented that LPS could induce autophagy mainly through four pathways as follows: (1) phosphorylation of p38 activates nuclear factor erythroid 2-related factor 2, resulting in the formation of aggresome-like induced structures and recruitment of LC3^20^,^24^,^37^; (2) induction of heme oxygenase-1^38^; (3) modulation of the ATG1 and protein B kinases (AKT) through Rab8a and PI3Kγ which are the upstream of mammalian target of rapamycin^21^; and (4) induction of ROS, which is important for MAPK pathways, including Jun N-terminal kinase (JNK) and ERK^22^,^39^. In this study, we demonstrated that A2AR inhibited LPS-induced neutrophils’ autophagy via activation of the PI3K-AKT pathway while repressing the ROS-JNK pathway. A2AR generally functions in the PKA and PKC pathways by stimulating the Gα subunit, but in our experiment, the inhibition of autophagy by A2AR was not regulated by these two classical mechanisms. Until now, there was no direct evidence indicating that A2AR could affect the AKT pathway in a Gα subunit-dependent manner. Several studies have demonstrated the phosphoinositide 3-kinase (PI3K)-AKT pathway could be regulated by the βγ G protein subunit (Gβγ) in pig neutrophils^40^, confirming that the Gβγ subunit was also functional *in vivo*. Moreover, mounting evidence has indicated that Gβγ participates in regulating the functions of neutrophils^41^,^42^,^43^. When the GPCR was activated, dissociated Gβγ could attract more p110γ,enhancing phosphorylation of AKT at the threonine 308 residue (T308) or serine 473 residue (S473)^44^. However, these studies were all concentrated on inhibitory GPCRs. Our results presented that activated A2AR enhanced the expression of p110γ significantly in neutrophils to mediate the PI3K-AKT pathway, which is the first confirmation that Gβγ from stimulatory GPCRs, such as A2AR, also has the biological functions. This not only broadens our understandings about A2AR, but also helps us to elucidate the functions associated with βγ G protein subunits.

Usually, both apoptosis and autophagy are recognized to be two fundamental biological processes to modulate cell survival and death. Multiple researches in some other cells are consistent with our finding that autophagy are not mutually exclusive but act in synergy or counter each other. The enhanced autophagy might protect cells from apoptosis^45^, or trigger the cell demise called autophagic cell death as well^46^. Several molecules have been shown important for modulating autophagy and apoptosis simultaneously, such as Atg5^47^, Bcl-2 protein^48^ and p53^49^. Especially Atg5, the basic molecule of autophagy machinery, could translocate from cytoplasm to mitochondria, triggering cytochrome c release and caspase activation^47^. Caspase family is important proteinase for apoptosis, and could be regulated by autophagy in previous studies^50^. However the downstream related molecules needs to be investigated. To further detect the mechanism of A2AR-inhibited autophagy suppressing apoptosis of neutrophils, we found blocking “caspase8-caspase3-PARP” signaling was involved in it. Generally, autophagy might modulate apoptosis in caspase dependent manner from two aspects: death receptors related pathways (caspase8) and mitochondria related pathways (caspase9)^51^. Finally the two ways both activate caspase3-PARP. Our results indicate that in neutrophils, autophagy modulates apoptosis via a Fas-associated exogenous pathways instead of a mitochondria-associated endogenous pathway, leading to the activation of caspase3 and PARP. Of note, Fas-associated exogenous pathways could also be activated by TNF-α, which suggests that the autophagy-regulated apoptosis in neutrophils may be mediated in an inflammation-dependent manner^52^. In addition, consistent with our results in mice, Pliyev B. K. *et al*. reported that A2AR could delay human neutrophils’ apoptosis^17^. However, they demonstrated that cAMP/PKA signaling axis is involved in this effect in human while we found it is not similar in mice. The differences may be resulted from (1) the different experimental conditions. They confirmed this phenomenon under normal conditions without any treatments or stimulations while we reiterated the conclusion in murine neutrophils with the LPS stimulation. (2) the differences between human and mouse neutrophils, Firstly, it is known that 50–70% of humans circulating leukocytes are neutrophils, whereas only 10–25% are in mice^53^. Thus we isolated the murine neutrophils from bone marrow, whereas the human neutrophils are usually isolated from peripheral blood. The different microenvironments might influence neutrophil’s response induced under treatment conditions. Secondly, lifespan of neutrophil in human is much longer than in mice. Generally, the half lifespan of circulating neutrophils is 1.5 hours and 8 hours in mice and human respectively^54^. This study also challenged that the average circulatory time of neutrophils could be 12.5 hours for mouse cells and even 5.4 days for human neutrophils under normal conditions^54^. The change of autophagy and apoptosis level in human might need longer time compared with our results confirmed in murine neutrophils. Thirdly, there are three types of granules in neutrophil: azurophilic (primary) granules, specific (secondary) granules and gelatinase (tertiary) granules. The components of granule in human and mice is a little different^55^. A study reported that the secondary granule participated in the formation of autophagosome in human neutrophil, leading to the vacuolization and apoptosis^56^, which indicated that different granule might influence autophagy and apoptosis in different way, which needs further investigation. Nevertheless, we can draw the conclusion that both cAMP/PKA-dependent and-independent manners could mediate the A2AR function on regulating neutrophil apoptosis in different situations.

Taken together, our experiments showed that activation of A2AR could suppress LPS-induced autophagy by inhibiting the ROS-JNK pathway as well as promoting the βϒ subunit dependent AKT-mTOR pathway. Meanwhile neutrophil apoptosis was regulated by autophagy, during which involved caspase8, caspase3 and PARP. These results not only increase our understandings of autophagy and apoptosis in neutrophils in response to systemic inflammation, but also identified a novel A2AR-mediated mechanism of modulating neutrophil survival during immune responses.

## Materials and Methods

### Wild type and A2AR knock-out mice

The A2AR^−/−^ mice were generated as previously described^30^. The mice were raised in a pathogen-free environment at the Animal Care Center of the Research Institute of Surgery and Daping Hospital (Third Military Medical University, Chongqing, China), with an libitum access to regular mouse chow and drinking water. All procedures used in this study were reviewed and approved by the Institutional Animal Care and Use Committee of Third Military Medical University and performed under the supervision of the facility veterinary staff. All methods were also performed in accordance with the relevant guidelines of Scientific Reports Editorial and publishing policies.

### LPS-induced SIRS model

SIRS mice models were achieved by intraperitoneal injection (i.p.) of LPS from *Escherichia coli* 0111:B4 (L2630, Sigma, Shanghai, China) dissolved with sterile 0.9% saline as previous did^19^. A dose of 11.25 million of EU/kg (10 mg/Kg) was used in these experiments. Onset of systemic inflammatory response occurs between 2–8 hours post-injection, and the signs of murine LPS-induced SIRS were recorded including body temperature and breath frequency. The severity score of murine SIRS were counted by 4 parameters as previously did^19^ after 6 hours and 24 hours of development: grooming behaviour (1-normal grooming, 2-reduced grooming and 3-no grooming), mobility (1-normalmobility, 2-partial impairment, 3-poormobility and 4-no mobility), piloerection (1-absence and 2-presence) and weeping eyes (1-absence and 2-presence). The scores of each parameters were summed as severity score index. Meanwhile, the mortality of each group (5 mice per group) was counted from the beginning to 24 hours. Then the survival lines were drawn.

### Primary isolation and identification of mice neutrophils from bone marrow

Primary neutrophil’s isolation from mouse bone marrow was performed as described previously^57^,^58^. The purity of morphologically mature neutrophils was 95–98% identified by marker of CD177 with flow cytometry (Becton Dickinson, USA). The attained neutrophils were diluted in RPMI-1640 media with 10% FBS to a final concentration of 10^6^/ml and cultured at 37 °C in an atmosphere of 5% CO_2_ for indicated time. The cell culture media Modified RPMI-1640 (SH30809.01B) was purchased from Hyclone Laboratories and Fetal Bovine Serum (FBS) was bought from ExCell Biology (FSS500, Shanghai).

### HL-60 culture and GFP-LC3 transfection

The cell line HL-60 (TCHU 23) was purchased from the Culture Collection of the Chinese Academy of Sciences, Shanghai, China. The cells were plated at 5 × 10^5^ per well cultured in IMDM with 20% FBS combined antibiotics (1:100) according to the recommendation on the official website of ATCC. HL-60 cells were treated with 1.25% DMSO for 4 days to acquire a neutrophil-like phenotype. The DMSO induced HL-60 was an approved model to study biological function of neutrophil as previous article showed^59^,^60^,^61^. Then electrotransfections were exerted through 4D-Nucleofector™ System (Lonza Cologne GmbH 50829 Cologne, Germany) combined with Nucleofector™ Solution under the recommendations of SF Cell Line 4D-Nucleofector™ X Kit. The transfection efficiency was more than 50% which was confirmed through positive plasmid control 0.4 μg pmaxGFP™ Vector and the cell viability (% trypan blue negative cells) is usually around 60 % after 24 hours. The LC3-GFP plasmid was kindly provided by Dr Zhenhong Ni in our department which was generated as described before^39^. The transfected cells were incubated in humidified 37 °C/5% CO_2_ incubator for 24 hours and then were treated for determined conditions and observed under the fluorescence microscope (Olympus IX81, Tokyo, Japan) with 40 lens.

### Western blot

The cells were treated as indicated conditions. And all the drugs were purchased as follows: Specific A2AR agonist CGS21680 (CGS), A2AR antagonist ZM24138 (ZM) and βγ- phosphatidylinositol kinase (PI3K) inhibitor Gallein (10 μM) were all bought from TOCRIS (Bristol, UK); highly selective PI3K-Akt inhibitor LY 294002 was bought from Cell Signaling Technology (Boston, MA); H-89 from Beyotime (Tianjin,China) or GF109203X (GFX) from SelleckChem (San Diego, CA) were used to inhibit protein kinase A (PKA) or protein kinase C (PKC) substrate phosphorylation and related cellular functions as previous^62^ (Both the working dose of the drugs are listed in the Supplementary Table 1). Subsequently, the whole-cell protein lysates were achieved and western blot analysis were performed as described previously^39^. Then the protein lysates were electroblotted on polyvinylidene difluoride (PVDF) membranes (Millipore,IPVH00010 and ISEQ00010), and probed with diluted primary antibodies (detailed dilution rates and detailed informations are as Supplementary Table 2 shows) overnight at 4 °C. Followed by related peroxidase (HRP) conjugated secondary antibodies, the proteins were developed and visualized using chemiluminescene substrate. auto radiographic film and imaging system. At final, the Blots were scanned and analyzed by Image J software. The normalized band intensities against corresponding GAPDH were calculated for precise comparison.

### Transmission electron microscope

Preparation of the ultrathin sections were described previously^63^. Briefly, fresh mouse neutrophils isolated from bone marrow were treated with or without LPS (1 μg/ml) for 2 hours. then washed and fixed in 4% glutaraldehyde. And post-fixation was finished by 2% osmium tetroxide. Thereafter, the neutrophils were dehydrated, treated with propylene oxide, and embedded. The ultrathin sections (65 nm) were subsequently stained with uranyl acetate and lead citrate and examined in a TECNAI-10 electron microscope.

### ROS generation and GSH detection

The intracellular ROS levels were measured by detecting the conversion of permeable ROS-dependent oxidation of 2′,7′-dichlorofluorescein discetate (DCFH-DA, Sigma, USA) to the fluorescent 2′,7′-dichlorofluorescein (DCF) with flow cytometry (FC) as previous study did^64^. The fluorescence intensity was expressed as the value of the ‘mean channel’, calculated by CellQuest software (Becton Dickinson, USA). The ROS inhibitor N-acetylcysteine (NAC) were used as negtive control^39^ of which were both obtained from Sigma-Aldrich (Shanghai, China). The GSH&GSSG detection kit (S0053,Beyotime, Tianjin, China) was used according to the instruction. The concentrations of total glutathione were detected by the absorbency at 412 nm and calculated by the standard curve. The GSH in samples were wiped off in order to achieve GSSG concentration. Finally the GSH concentrations were gained through total glutathione minus GSSG.

### Analyses of caspase 8 and caspase 9 activity

The caspase 8 and caspase 9 detection kits (BB4107&BB4108) were purchased from Bestbio technology (Shanghai, China) and detailed operations were referred to the previous article^65^.

### Apoptosis analyses

Apoptosis was assayed by Muse^®^ Annexin V & Dead Cell Assay (MCH100105, Merck Millipore, Darmstadt, Germany). The apoptosis detections of blood smears were achieved by Apoptag Plus Fluorscein *in situ* Apoptosis Detection Kit (S7111, Millipore, Temecula, USA) according to the instruction. Rapamysin (RAPA) and 3-MA purchased from SelleckChem (San Diego, CA) were utilized to enhance or recede autophagy level according to the previous papers^39^,^66^.

### Statistical analyses

Statistical comparisons of more than two groups were performed by A one-way analysis of variance (ANOVA). The student’s t-test was utilized for statistical evaluation of differences between experimental groups. Graphic data represent the mean ± SEM. A value of p < 0.01 was considered statistically significant. All statistical analyses were performed using Graph Pad Prism 4.03 (GraphPad Software, San Diego California USA).

## Additional Information

**How to cite this article**: Liu, Y.-W. *et al*. Activation of Adenosine 2A receptor inhibits neutrophil apoptosis in an autophagy-dependent manner in mice with systemic inflammatory response syndrome. *Sci. Rep.* **6**, 33614; doi: 10.1038/srep33614 (2016).

## Supplementary Material

Supplementary Information
