# Supplementary material for: Activation of Adenosine 2A receptor inhibits neutrophil apoptosis in an autophagy-dependent manner in mice with systemic inflammatory response syndrome
Source: Sci Rep. 2016 Sep 20;6:33614. doi: 10.1038/srep33614 (PMC5028892; doi:10.1038/srep33614)
Supplement: Supplementary Information [file srep33614-s1.pdf]

Activation of Adenosine 2A receptor inhibits neutrophil apoptosis in an autophagy-dependent manner in mice with systemic inflammatory response syndrome

Yang-Wuyue Liu[1]; Ting Yang[1]; Li Zhao[1]; Zhenhong Ni[1]; Nan Yang[2];

Fengtian He[1] ; Shuang-Shuang Dai[1*]

Supplementary Table S1: Abbreviations and working doses about drugs used in this article

Chemical Ingredient	Abbreviation	Working dose	Company
Lipopolysaccharides from *Escherichia coli 0111:B4*	LPS	1000ng/ml	Sigma-Aldrich (Shanghai, China)
N acetylcysteine	NAC	10mM	
3-[4-[2-[[6-amino-9-[(2R,3R,4S,5S)-5-(ethylcarbamoyl)-3,4-dihydroxy-oxolan-2-yl]purin-2-yl]amino]ethyl]phenyl]propanoic acid	CGS	0.1 μ M	TOCRIS (Bristol, UK)
4-(2-[7-Amino-2-(2-furyl)[1,2,4]triazolo[2,3-a][1,3,5]triazin-5-ylamino]ethyl)phenol	ZM	1 μ M	
3',4',5',6'-Tetrahydroxyspiro[isobenzofuran-1(3H),9'-(9H)xanthen]-3-one	Gallein	10 μ M	
Highly selective inhibitor of phosphatidylinositol kinase	LY294002	10 μ M	Cell Signaling Technology (Boston, MA)
N-[2-[[3-(4-bromophenyl)-2-propenyl]amino]ethyl]-5-isoquinolinesulfonamide dihydrate dihydrochloride	H-89	10 μ M	Beyotime (Tianjin,China)
1H-Pyrrole-2,5-dione,3-[1-[3-(dimethylamino)propyl]-1H-indol-3-yl]-4-(1H-indol-3-yl)	GFX	5 μ M	SelleckChem (San Diego, CA)
Rapamysin	RAPA	50nM	
3-methyladenine	3-MA	2mM	

Supplementary Table S2: Dilution ratios and companies about antibodies used in this paper.

Antibody	Dilution	Company
LC3	1:1000	Cell Signaling Technology(Boston, MA)
P38		
p-P38		
p-ERK		
p-JNK		
PARP		
Cleaved caspase3		
P110	1:800	Santz Cruz Biotechnology (Dallas, Texas)
P110γ		
AKT		
p-AKT(Thr 308		
p-AKT(Ser 473)		
goat anti-rabbit horseradish peroxidase conjugated antibody	1:10000	KangChen Biotechnology(Shanghai,China).
goat anti- mouse horseradish peroxidase conjugated antibodies		
donkey anti-goat horseradish peroxidase conjugated antibodies		
GAPDH	1:3000	
